# The necessity of enhancing menstrual health policy in Iran: A letter to editor

**DOI:** 10.18502/ijrm.v23i4.18787

**Published:** 2025-06-10

**Authors:** Maryam Gharacheh, Fahimeh Ranjbar

**Affiliations:** Nursing and Midwifery Care Research Center, Health Management Research Institute, Iran University of Medical Sciences, Tehran, Iran.

##  Dear Editor,

Menstrual policies across the globe encompass a range of initiatives, laws, and practices aimed at addressing the menstrual health and hygiene requirements of individuals who menstruate, while also ensuring their rights and well-being. Numerous countries have introduced programs to educate people about menstruation, dispel myths and taboos, and promote menstrual hygiene practices. These educational efforts often target girls in schools, community settings, and public awareness campaigns. In certain regions and countries, policies have been implemented to enhance affordability and accessibility of menstrual products, and in some cases, even provide them for free (1). Furthermore, in some nations, female employees are entitled to take paid leave for severe menstrual-related pain or discomfort as part of efforts to promote gender equality and protect women's health. While not widely implemented, specific countries such as Japan, Taiwan, China, South Korea, Indonesia, Zambia, and Mexico have laws or guidelines allowing for menstrual leave (2). Furthermore, various countries have eliminated taxes on menstrual products, aiming to reduce the financial burden associated with acquiring these essential hygiene items. Examples of such countries include Kenya, Canada, Australia, India, Colombia, Malaysia, Nicaragua, Jamaica, Nigeria, Uganda, Lebanon, Trinidad, and Tobago, and several states in the United States (3).

It is crucial to recognize that menstrual policies vary significantly among countries, influenced by cultural, social, and economic factors. Despite some progress, many regions still need to address the stigma surrounding menstruation and implement comprehensive policies that promote menstrual health and well-being (4). It is a matter of concern that Iran lags in recognizing the significance of comprehensive menstrual health policies and laws. The absence of formal and comprehensive menstrual education in schools or public settings in Iran has left numerous girls uninformed or misinformed about this natural process (5). In Iran, menstrual education typically occurs within families, with maternal figures often responsible for imparting knowledge about menstruation to their daughters. This education often emphasizes concepts such as privacy and modesty. However, cultural practices and taboos surrounding menstruation can result in negative societal perceptions, leading to stigma, shame, or discrimination against many women and girls (6).

While menstrual products such as sanitary pads and tampons are readily available in Iran, menstrual cups are a recent introduction to the Iranian market. A cross-sectional study highlighted high levels of acceptance and safety perceptions among Iranian women regarding menstrual cups, with users expressing significant satisfaction and recommending the product to others (7). Unlike some countries, Iran currently lacks specific laws or regulations that grant menstrual leave or address taxation on menstrual products. Consequently, sanitary pads, tampons, and menstrual cups are subject to a value-added tax, impacting their affordability. Policies ensuring the accessibility and affordability of menstrual hygiene products are essential to safeguarding the well-being of women and girls in Iran. However, the percentage of Iranian women who have limited access to menstrual products, particularly students, marginalized, homeless, or incarcerated women, remains unknown. Therefore, accurate data is essential for effective policy-making in this area.

In recent years, Iran has focused on population growth policies through legislation like the law on population youth and family support (8). While this legislation addresses certain aspects of women's reproductive health, the oversight of menstrual health is evident. Integrating menstrual policies into population policies is recommended, as access to menstrual products and education can have a significant impact on fertility rates and reproductive health outcomes. Ensuring all menstruating individuals have access to adequate care and basic needs is a human rights issue that demands attention. Inadequate menstrual hygiene can have detrimental effects on both physical and mental health, including increased risks of infections, school absenteeism, low academic performance, and perpetuation of menstrual cycle stigma (9). The absence of specific menstrual health policies in Iran, coupled with a lack of educational reform and awareness campaigns, exacerbates gender inequalities and violates the fundamental rights of women and girls. The integration of menstrual policies into population policies is vital to advancing gender equality and fulfilling human rights obligations (10). By prioritizing the rights and needs of women and girls in Iran, ensuring their access to accurate information, affordable hygiene products, and supportive environments, the government can take significant steps toward promoting well-being, reducing stigma, and promoting gender equality.

##  Conflict of Interest

The authors declare that they have no competing interest.
